# Quantitative Modulation of PpIX Fluorescence and Improved Glioma Visualization

**DOI:** 10.3389/fsurg.2019.00041

**Published:** 2019-07-16

**Authors:** Michael Reinert, Deborah Piffaretti, Marco Wilzbach, Christian Hauger, Roland Guckler, Francesco Marchi, Maria Luisa D'Angelo

**Affiliations:** ^1^Laboratory for Biomedical Neurosciences, Neurocenter of Southern Switzerland, Ente Ospedaliero Cantonale, Torricella-Taverne, Switzerland; ^2^Department of Neurosurgery, Neurocenter of Southern Switzerland, Ente Ospedaliero Cantonale, Lugano, Switzerland; ^3^Faculty of Biomedical Neurosciences, Università Della Svizzera Italiana, Lugano, Switzerland; ^4^Medical Faculty, University of Bern, Bern, Switzerland; ^5^Faculty of Medicine, Graduate School for Cellular and Biomedical Sciences, University of Bern, Bern, Switzerland; ^6^Carl Zeiss Meditec AG, Oberkochen, Germany

**Keywords:** GBM—glioblastoma multiforme, 5-ALA=5-aminolevulinic acid, protoporphyin IX, quantification, breakdown, visualization, microscope

## Abstract

5-Aminolevulinic acid (5-ALA) induced fluorescence to augment surgical resection for high grade glioma has become a standard of care. Protoporphyrin IX (PpIX) visibility is however subject to the variability of the single tumor expression and to the interobserver interpretation. We therefore hypothesized that in different glioma cell lines with variable 5-ALA induced fluorescence, the signal can be pharmacologically increased. We therefore analyzed in three different GBM cell lines, with different expression of epidermal growth factor receptor (EGFR), the variability of 5-ALA induced PpIX fluorescence after the pharmacological blockade at different steps of PpIX breakdown and influencing the outbound transport of PpIX. Using flow cytometry, fluorescence microplate reader, and confocal microscopy the PpIX fluorescence was analyzed after exposure to tin protoporphyrin IX (SnPP), deferoxamine (DFO), and genistein. We furthermore constructed a microscope (Qp9-microscope) being able to measure quantitatively the concentration of PpIX. These values were compared with the extraction of PpIX in tumor biopsy taken during the GBM surgery. Although all three cell lines showed an increase to 5-ALA induced fluorescence their baseline activity was different. Treatment with either SnPP, DFO and genistein was able to increase 5-ALA induced fluorescence. Qp9-microscopy of tumor sample produced a color coded PpIX concentration map which was overlaid on the tumor image. The PpIX extraction from tumor sample analyzed using the plate reader gave lower values of the concentration, as compared to the expected values of the Qp9-microscope, however still in the same decimal range of μg/mL. This may be due to homogenization of the values during extraction and cell disaggregation. In conclusion pharmacological augmentation in GBM cell lines of PpIX signal is possible. A quantitative PpIX map for surgery is feasible and may help refine surgical excision. Further correlations of tumor tissue samples and Qp9-microscopy is needed, prior to develop an intraoperative surgical adjunct to the already existing 5-ALA induced surgery.

## Introduction

**Figure 1:**
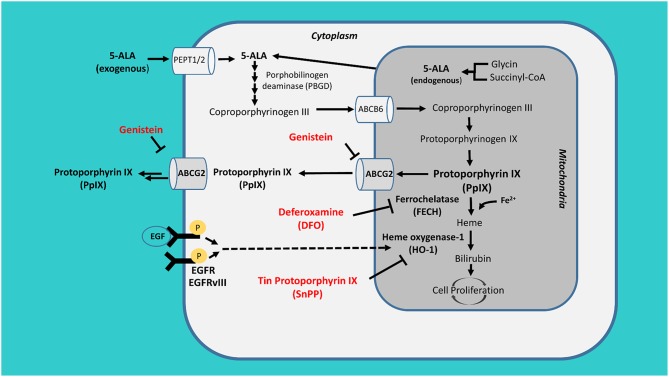
PpIX metabolism. Exogenous 5-aminolevulinic acid (5-ALA) is internalized by tumor cells through the peptide transporter 1 and 2 (PEPT1/2), it is then converted into the fluorescent tracer protoporphyrin IX (PpIX) inside the mitochondrion. The accumulation of PpIX is antagonized by its active conversion through ferrochelatase (FECH) and heme oxygenase-1 (HO-1) and its efflux through ATP-binding cassette sub-family G member 2 (ABCG2). These proteins could be blocked by the action of deferoxamine (DFO), tin protoporphyrin IX (SnPP), and genistein.

It is widely accepted that the extent of surgical resection plays an important role in overall survival in patients with glioma, both in IDH wild type and IDH mutated, and both in high grade and low-grade glioma (1, 2). In high-grade glioma, the extent of resection is guided by MRI and directed to the contrast-enhancing portion of the lesion, while in low-grade glioma the resection is decided based on the tumor infiltration shown by T2 sequence alteration (3, 4). New techniques as fluorescence have become a standard of care for intraoperative resection of high grade glioma (4). Nowadays, however, growing evidence support the use of these detection techniques also in low grade glioma (5). PpIX fluorescence and its concentration in tumor cells depends on the balance between its synthesis, catabolism and outflow. The amount of PpIX in tumor cells is favored by both the activity of cytosolic porphobilinogen deaminase (PBGD) during the replication phase, and by the down-regulation of ferrochelatase (FECH) (6). However, other factors such as protein transporters, tyrosine kinase activity and its downstream effect on hemoxygenase-1 (HO-1), availability of free Fe^2+^-ions and its effect on FECH, are related with PpIX fluorescence (7, 8). Because of the above mentioned factors, the final enhancing results are highly variable and difficult to predict both in clinical and laboratory setting, and might also depend on EGFR expression status in glioma cell lines (7). Proteins transporters such as ATP-binding cassette subfamily G member 2 (ABCG2) regulate intracellular concentration of PpIX. These proteins may also be differently expressed or activated in relation with the epigenetic status of GBM (9) (Figure 1).

Further well-known factors such as 5-ALA patient administration concentration, exposure time and tumor cell concentration influence the concentration of PpIX at the local level (4, 10). Furthermore, fading requires also consideration during light exposure. Also, 5-ALA fluorescence may vary spatially from one area to another of GBM (11). Lastly, two further factor that limit the validation of PpIX fluorescence consist in the inter-observer variability and in the different signal imaging obtained by the microscopic view or by the video screen. Based on all above mentioned limitations, there is an urgent need to go beyond the visual capacity of the human eye by measuring more accurately the tumor borders with quantitative pixel based method, and possibly by establishing a definite threshold for tumor activity presence.

The overall purpose of the study was to better understand the metabolic pathway of PpIX, and thereafter to augment pharmacologically its signal in different GBM cell lines in order to increase its visibility. This achievement might be exploited in the future also in photodynamic therapy. We thus selected three compounds: tin protoporphyrin IX (SnPP) a HO-1 blocker, deferoxamine (DFO) an iron chelator, and genistein. Genistein is a natural product (Isoflavone) (12), known as an angiogenesis inhibitor and as an inhibitor of ABCG2 transporter protein, thus influencing the outbound cell traffic of PpIX. All compounds are either FDA approved or previously described in human use. In order to avoid the limit of inter-observer variability and to capture also low concentrations of fluorescence tracer, barely visible by naked eye, a quantification of PpIX during surgical resection is required. We therefore developed a quantitative PpIX microscope (Qp9) according to previously described setup (13–15) aiming to correlate, for the first time, the color coded matrix images with the effective concentration of PpIX is to correlate these matrix images with the effective extraction concentration of PpIX.

## Methods

### Cell Culture

Human GBM cell line U87MG obtained from American Type Culture Collection (89081402-1VL, Sigma-Aldrich) was maintained in Dulbecco's Modified Eagle Medium (DMEM, 61965, Gibco® Life technologies Europe), GlutaMAX cell culture medium supplemented with 10% fetal bovine serum (FBS, 10270, Gibco® Life technologies Europe), 1% non-essential amino acids (MEM NEAA, 11140, Gibco® Life technologies Europe), 1 mM sodium pyruvate (S8636, Sigma-Aldrich), penicillin 10,000 units/mL and streptomycin 10,000 μg/mL (15140, Gibco® Life technologies Europe).

The human GBM cell line U87wtEGFR overexpressing the EGFR gene, was generously provided by Prof. Dr. Frank Furnari (Laboratory of Tumor Biology, Ludwig Institute for Cancer Research, University of California-San Diego) and was cultured in DMEM GlutaMAX, 10% FBS and 1% penicillin-streptomycin, supplemented with 100 μg/mL of G418 disulfate salt (A1720, Sigma-Aldrich). U87MG vIII 4.12 cells stably expresses high level of the mutant EGFR variant III (deletion of exons 2-7) (CL 01004-CLTH, Tebu-bio) were maintained in DMEM GlutaMAX, 10% FBS, 1% penicillin-streptomycin and 0.2% of gentamicin 10 mg/mL (15710049, Thermo Fisher Scientific, Life Technologies Europe), enriched with 100 μg/mL of G418. All cells were kept at 37°C, 5% CO_2_ atmosphere, in static conditions. Cells were harvested by incubation for 5 min at 37°C with 500 μL TrypLE™ Express Enzyme (1X), no phenol red (12604021, Gibco® Life technologies Europe) and blocked with DMEM supplemented with 10% FBS. During the time of the experiment, the cells were plated and after 24 h the medium was replaced with serum-free DMEM, high glucose, HEPES, no phenol red (21063045, Gibco® Life technologies Europe) for at least 24 h before starting the treatment (16).

### Drug Treatment

5-ALA (Fagron DAC 2011), was freshly dissolved in milli-Q water at an intermediate concentration of 1 M and then diluted in serum free medium at a final concentration of 1 mM. DMSO 0.5% (vol/vol) (D2650, Sigma-Aldrich), was added to 5-ALA to increase its permeability into the cells as previously demonstrated by Lawrence et al. (17). Deferoxamine mesylate (492880, Desferal®, DFO, Novartis) was dissolved in milli-Q water. SnPP (sc-203452, Santa Cruz Biotech) and genistein (sc-3515, Santa Cruz Biotechnology) were dissolved in DMSO (16).

### PpIX Evaluation by Flow Cytometric Analysis

U87 cells were plated in a 6-well plate and treated with DFO 100 μM, SnPP 100 μM, or genistein 25 μM alone or in combination with 5-ALA at 1 mM or with all combinations of these treatments. After 8 h of treatment, cells were harvested, washed twice with phosphate buffered saline (PBS, 10010056, Gibco® Life technologies Europe) and centrifuged at 2,000 rpm for 5 min. The pellet was resuspended in 100 μL of PBS and the single cells suspension was analyzed by flow cytometry (excitation 408 and emission 630/38; CytoFLEX S, flow cytometry-Beckman Coulter) (16).

### Confocal Microscopy

Cells were plated into Ibidi μ-slides VI0.4 (80606, Ibidi). After 24 h in FBS-free medium, cells were treated with DFO 100 μM, SnPP 100 μM, or genistein 25 μM alone or in combination with 5-ALA at 1 mM or with all combinations of these treatments. After 8 h of treatment, cells were washed twice with PBS, fixed with 4% paraformaldehyde for 10 min, washed with PBS. Slides were examined with a confocal line scanning microscope (LSM) Leica TCS SP5 equipped with the objective HCX PL APO lambda blue 40.0 ×1.25 OIL UV and an excitation of 405 nm and an emission of 620–650 nm (16).

### Operative Microscope and Quantitative PpIX Microscope

**Figure 2:**
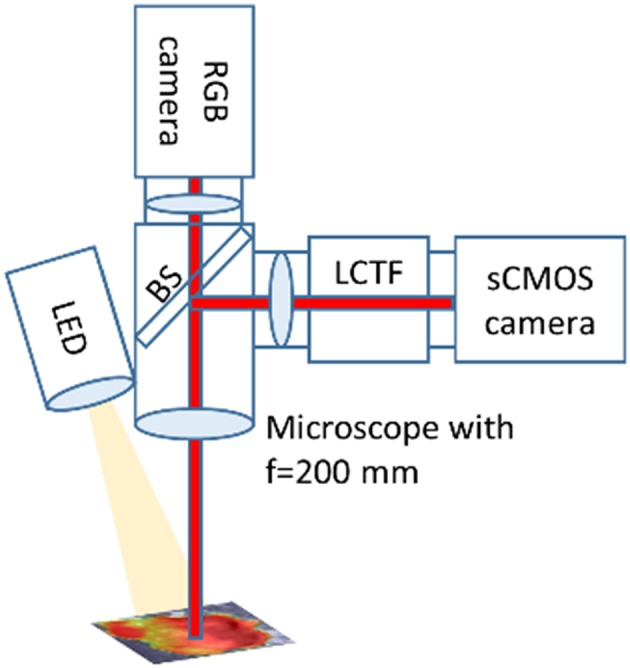
Quantitative PpIX microscope. A tissue sample or cell culture is illuminated by a LED light source with white light or light at 405 nm. The microscope collects the reflected and fluorescent light with an objective lens with f = 200 mm. A beam splitter (BS) splits the light into two paths. One path is imaged with a hyperspectral camera setup. For an overview the second path is imaged on a color camera (RGB camera). The hyperspectral setup consists of a scientific CMOS camera (sCMOS) and a liquid crystal tunable filter (LCTF).

For our tumor resection and cryosectioned slice of GBM xenograft mouse model, an operating microscope (OPMI) Pentero 900 (Carl Zeiss Meditec, AG, Oberkochen, Germany) with a BLUE-400 mode was used for imaging (8-bit RGB, 1,920 ×1,080 pixels) at a 20-cm distance from the target (18). Operative video was registered on BrainLab Buzz for later analysis (**Figure 4**, Video S1). For the quantification of the tumor samples taken during the surgery we constructed our custom-made microscope (Qp9) (Figure 2) its basic functionality described previously by Valdes et al. (13–15). The Qp9 microscope was calibrated to brain tissue mimicking phantoms with different known PpIX concentrations. The tumor samples were taken (Video S1) and then frozen at −80°C for later analysis with color matrix images obtained with Qp9. The sample was processed to trypLE lysis for cell disaggregation, as previously described in cell culture section, and then PpIX was extracted for TECAN analysis, for comparison with the Qp9 microscope.

#### Qp9 Microscope Setup

Tissue samples and cell cultures are imaged with a hyperspectral camera setup and a color camera attached to a microscope (OPMI pico, Carl Zeiss Meditec) with a fixed working distance of 200 mm. The image is divided by a beam splitter between the two imaging paths. The LED light source is capable to produce white light and violet light (405 nm) with an intensity of up to 30 mW/cm^2^ at the sample.

The hyperspectral setup consists (13) of a scientific CMOS (pco.edge 4.2, PCO AG) camera and a liquid crystal tunable filter (LCTF, Meadowlark Optics, Inc.) (13–15).

The LCTF can be tuned between 420 and 730 nm in one nanometer steps. A typical full width at half maximum (FWHM) of a passband is 12 nm with a transmittance in the range of 5–30%. Outside the passband the transmittance is smaller than 1%.

A standard color camera (uEye CP with IMX252, IDS imaging GmbH) is connected to generate overview images.

With the hyperspectral setup one can record hyperspectral image stacks. The reflectance of the sample can be recorded using white light from the LED light source and fluorescence hyperspectral image stacks can be recorded using 405 nm light from the LED light source. For each pixel a white light reflectance and fluorescence spectrum is available.

These spectra are used to calculate absolute PpIX concentrations (15). So far, the system is calibrated to PpIX brain mimicking phantoms. The fluorescence spectrum is spectrally unmixed, so the pure PpIX signal can be separated from autofluorescence and other background signals such as ambient light. Then the white light spectrum is used to normalize for different tissue properties (different scattering and absorption).We get a PpIX concentration map of the measured samples overlaid on the image of the sample.

#### Calibration

For calibrating the system, tissue phantoms with defined optical properties were created to mimic brain tissue (19). PpIX was dissolved in dimethyl sulfoxide to get different concentrations (10 ng/ml−5 μg/ml), intralipid and yellow food colorant were used to generate varying scattering (reduced scattering coefficient at 635 nm, μ'_sm_ = 8.7–14.5 cm^−1^) and absorption properties (absorption coefficient at 405 nm, μ_ax_ = 20–60 cm^−1^). The lower detection limit of the system at one second integration time is 10 ng/ml of PpIX.

## Results

### Inhibition of PpIX Metabolism of HO-1 by Tin Protoporphyrin IX (SnPP)

**Figure 3:**
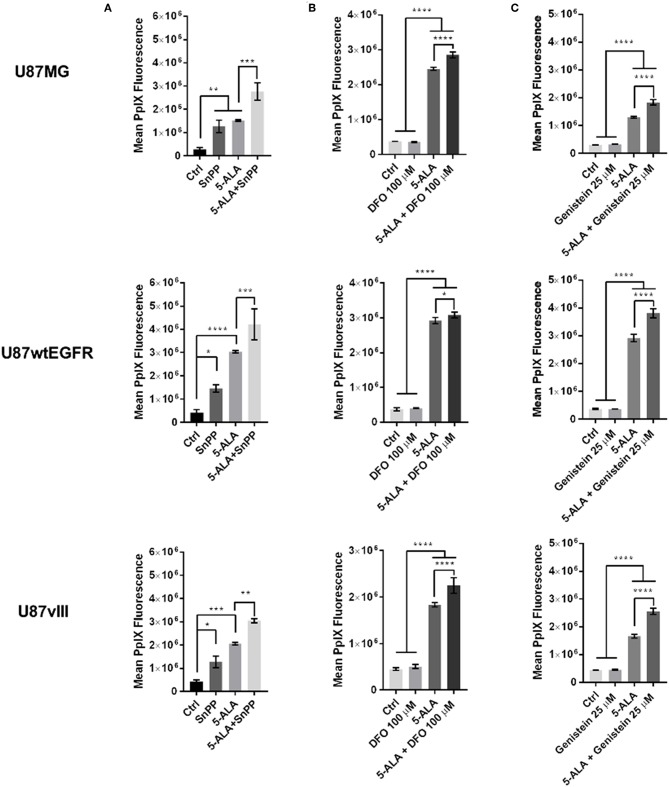
Effect of single treatments on U87 GBM cell lines. Effect of **(A)** tin protoporphyrin IX (SnPP), **(B)** deferoxamine (DFO), and **(C)** genistein on three U87 cell lines differing for EGFR expression. Control untreated cells are compared with cells treated with the selected compound alone and cells treated with 5-ALA alone or with combination of 5-ALA and the indicated drug. Graphs show that all the three treatments in combination with 5-ALA significantly induce the fluorescence compared to 5-ALA alone. Results are expressed as mean ± SD (one-way ANOVA, Sidak's multiple comparison test *p < 0.05; **p < 0.01; ***p < 0.001; ****p < 0.0001). Where not specified the difference is not significant.

Having previously established the final concentration of 5-ALA at 1 mM for 8 h as the optimal conditions for 5-ALA treatment and quantification of PpIX fluorescence (16), we then proceeded to study the effect of drugs that modulate proteins involved in PpIX conversion into non-fluorescent metabolites. Dose response and cytotoxicity analysis for SnPP showed that the best compromise between PpIX accumulation and cytotoxicity is a concentration of SnPP at 100 μM (16). So, we treated 2 ×10^5^ adherent cells/well with SnPP at 100 μM alone or in combination with 5-ALA for 8 h and then determined the mean cellular PpIX fluorescence by flow cytometry for biological duplicates. Based on two independent experiments performed by flow cytometry, we showed that the HO-1 inhibitor SnPP is able to significantly improve 5-ALA-induced PpIX fluorescence in U87MG and U87vIII, alone or in combination with 5-ALA. SnPP and 5-ALA co-treatment augmented the mean PpIX fluorescence by 81% for U87MG cells, by 39% for U87wtEGFR cells and by 48% for U87vIII cells compared to cells treated with 5-ALA alone, set as 100% (Figure 3A). The SnPP treatment alone showed an increment of PpIX fluorescence by 358% for U87MG cells, 252% for U87wtEGFR cells and 198% for U87vIII compared to untreated cells, set as 100% (Figure 3A) (16).

### Inhibition of PpIX Synthesis by Fe^2+^-Chelation and Inhibitory Effect on FECH

Based on our previous studies on cell cytotoxicity, we selected DFO at 100 μM to test the effect of FECH inhibition for 8 h in the presence or absence of exogenous 5-ALA (16). The treatment with DFO alone compared to control untreated cells showed a non-significant variation in PpIX fluorescence. In contrast, in the presence of exogenous 5-ALA, DFO significantly improved 5-ALA-induced PpIX fluorescence in all the three cell lines. In detail, the increase was of 16% for U87MG, of 6% for U87wtEGFR and of 22% for U87vIII cells when compared to cells incubated with 5-ALA alone, set as 100% (Figure 3B) (16).

### Inhibition of PpIX Efflux by Genistein Acting on ABCG2

To retain the PpIX accumulated inside the cells, we tested the effect of inhibiting the main PpIX efflux transporter ABCG2 with genistein. Based on our previous cytotoxicity and pharmacological studies, we selected a genistein concentration of 25 μM (16). With the selected concentration we performed a more detailed analysis in biological triplicates of PpIX fluorescence by flow cytometry in the absence or presence of exogenous 5-ALA. Endogenous PpIX fluorescence was not affected by the blockade of the ABCG2 transporter in presence of genistein, whilst this was the case when 5-ALA was added to the culture medium for the three cell lines. In fact, the combined treatment with genistein and 5-ALA compared to 5-ALA alone (set as 100%) increased PpIX fluorescence by 42% for U87MG cells, by 31% for U87wtEGFR cells and by 54% for U87vIII cells (Figure 3C) (16).

### Combined Treatments Improves PpIX Accumulation in U87 Glioblastoma Cell Lines

**Figure 4:**
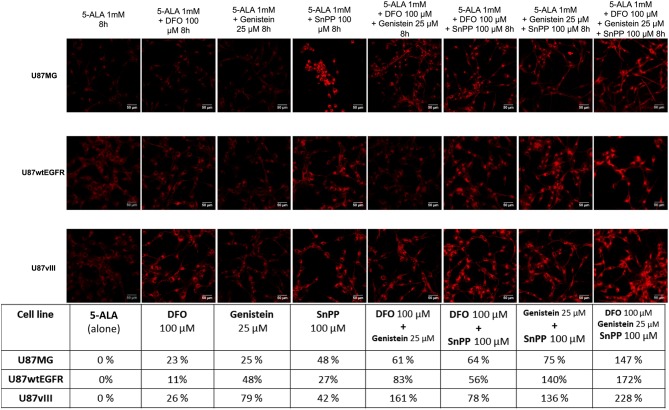
PpIX fluorescence accumulation after single and combined treatments. Confocal images showing the increment in PpIX fluorescence (represented in red, excitation 405 nm and emission 635 nm) in GBM cells after single and combined treatment with two or three drugs compared to 5-ALA alone (represented as 0%). Scale bars represent 50 μm. Table summarizes the increment of PpIX fluorescence in percentage. DFO (deferoxamine), SnPP (tin protoporphyrin IX).

Detailed flow cytometric analysis led to the identification of combined treatments composed of two drugs leading to significantly increased mean PpIX fluorescence. For all three cell lines, the combination of reduced PpIX metabolism and impaired PpIX efflux resulted in augmented 5-ALA-induced fluorescence. Whilst SnPP and genistein is the best combination for U87MG (75% increase compared to 25% with genistein alone or 48% for SnPP alone) and U87wtEGFR cells (140% increase compared to 48% for genistein and 27% for SnPP), U87vIII cells responded better to DFO combined with genistein (161% increase compared to 79% with genistein and 26% for DFO) in the presence of exogenous 5-ALA, set as 0%. Maximal PpIX fluorescence is observed for the three lines with the combination of all three drugs, indicating that neither SnPP nor DFO, when tested alone, are able to completely block PpIX metabolism. In the presence of the three drugs the increment in PpIX fluorescence reached 147% for U87MG cells, 172% for U87wtEGFR cells and 228% for U87vIII cells as reported in the table of Figure 4. These data were confirmed by qualitative confocal microscopy analysis (Figure 4). Overall, these data indicated that inhibition of PpIX metabolism coupled to inhibition of PpIX efflux is expected to improve the visualization of GBM cells. Nevertheless, the optimal drug combination may depend on the genotype of the GBM cell, in particular considering the presence of defined gene mutations.

### Intraoperative PpIX Fluorescence Guided Tumor Resection and Postoperative Quantification of PpIX Fluorescence of Tumor Samples and Comparison With PpIX Extraction

**Figure 5:**
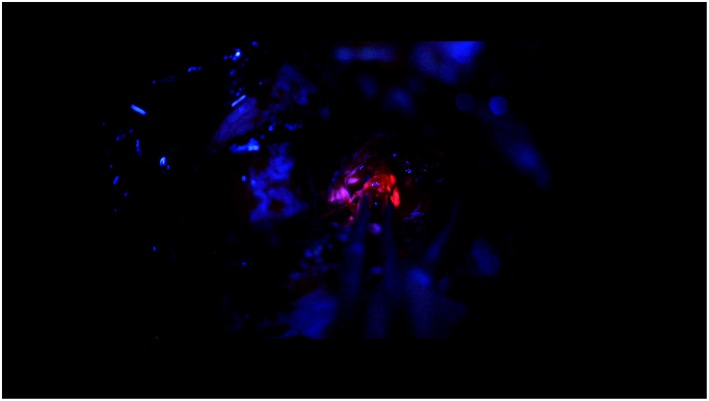
5-ALA guided surgery with OPMI PENTERO 900. Image of video showing the surgical intervention of glioblastoma removal. Under the blue light the tumor bulk is red, the fluorescence in the tumor margin is pink and more vague.

**Figure 6:**
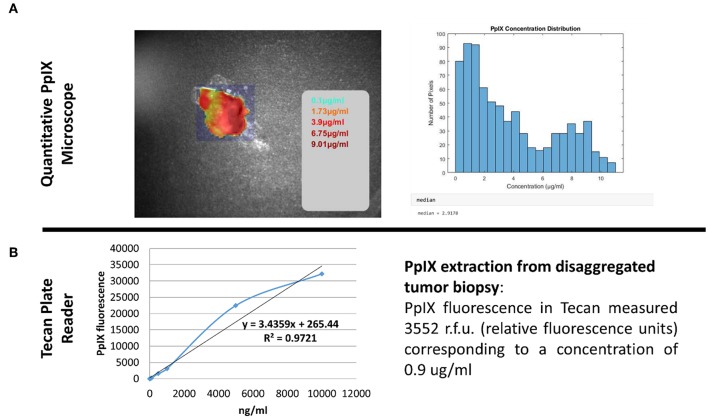
Quantitative PpIX measurement. Comparison of two different methods able to quantitatively measure the accumulated PpIX in tissue biopsy. **(A)** Shows quantitative measurement performed with the Qp9 Zeiss microscope whereas **(B)** shows results obtained after methanolic-perchloric acid extraction from the whole biopsy.

Figure 5 and Video S1 show the intraoperative resection of GBM and the tissue which has been taken for later analysis with the Qp9-microscope. Figure 6A shows the color-coded image of the tumor samples with the concentration values for PpIX. The color-coded image represents areas of high and low PpIX concentration on its surface. The automated area of investigation of the Qp9 microscope (Figure 6A) contains areas outside or at the margin of the tumor specimen and such are practically reported as very low or absence of PpIX (<0.1 μg/ml). This however does not affect the correlation as these very low values are not considered in the sample extraction. Tumor sample has thereafter been disaggregated and analyzed for its PpIX concentration (Figure 6B). The values of the disaggregated tumor tissue show concentration below the expected values measured by the Qp9 microscope yet in the same decimal.

## Discussion

There is no doubt that 5-ALA induced fluorescence has an impact on augmentation of the surgical resection in GBM surgery (20). Additionally, 5-ALA induced fluorescence harbors the potential for photodynamic therapy. For both treatments, a selective and controlled augmentation and especially quantification of the PpIX fluorescence signal is crucial for developing the technologies further. Therefore, we studied, in three different cell lines, the optimal conditions and pharmacological possibilities to augment the signal by influencing the balance of production, consumption and outbound transportation of PpIX. We also identified that different GBM cell lines have different PpIX signal, a phenomenon which has been also observed in the clinical situation (21). The evaluation of PpIX fluorescence positivity during the surgical operation of GBM is especially subjective to the surgeon's eye, limiting the usefulness and thus the impact on resection. Hence user independent evaluation of the operation situs will become necessary. Different groups in the past have described offline imaging techniques or online probes to interpret the quantitative values of PpIX (22). Yet the verification of microscopic arbitrary values as measured in the past and as in our study by the Qp9 microscope need to be further verified by selective tumor tissue analysis and quantitative PpIX extraction.

**Figure 7:**
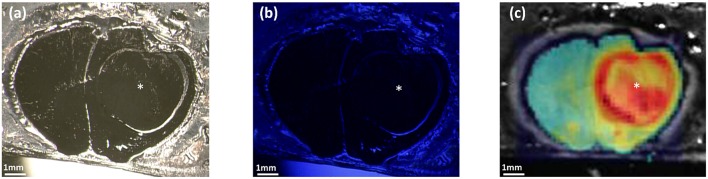
Comparison between OPMI PENTERO 900 and Qp9 Zeiss microscopes. The figures show cryosectioned slice of GBM xenograft mouse model. In **(a,b)** visualization under OPMI PENTERO 900 with white light source and UV laser Blue 400, respectively. **(c)** Shows the color coded matrix image obtained after the PpIX quantification by Qp9. *tumor region. Scale bars = 1 mm.

The values measured after extraction of PpIX from tumor samples were lower than what the values were in the Qp9 analysis. Reasons for that may be divers, resulting from extraction dilution or the disaggregation process. Although approximation of the effective PpIX concentration might technically be possible in the real surgical resection situation, these quantitative values will probably have no effect on the decision making for the tumor resection itself. Furthermore, during surgery of human GBM different factors may influence the visibility, respectively, the detection of PpIX fluorescence both with the naked eye or with the Qp9 microscope. Factors such as bleaching or different focus distance or even fluid covering may influence the signal intensity. Therefore, a sensible detection technique such as Qp9 or a mechanism enhancing the signal intensity such as heme oxygenase-1 blocker, iron chelating agents or PpIX transporter protein blocker may possibly be combined in the future for more sensible detection of different fluorescenting GBM or maybe even lower grade glioma. These findings will however need to be correlated with effective tumor cell infiltration in brain tumor. We have previously demonstrated that different GBM cell lines may have different PpIX signal intensity when treated with the same parameters (7, 16), a phenomenon also described *in vivo* (21). This phenomenon can be also seen in our mice GBM tumor model, as demonstrated in Figure 7, where tumor not visible to the naked eye with the conventional blue light of the operation microscope, can be made visible with color coded matrix under the Cp9 microscope. Belykh et al. have previously analyzed three different techniques (Scanning fiber endoscope (SFE), blue light and confocal scanning microscope) and concluded that SFE provides new endoscopic capability to visualize PpIX positivity at cellular levels (18, 23). We agree with Belykh et al. (18) that a more refined technique for visualization of PpIX such as SFE or now Qp9 are necessary. Techniques are complementary and probably cannot replace one with the other. The advantage of Qp9 over SFE may be that it can give the surgeon a faster overall picture of where remaining tumor is to be searched for and resected, however at the fate of more artifacts such as fluids, bleaching and focus distance. SFE will probably result more locally precise. Therefore, possibly a combination of both techniques may prove to be even more effective. Our next steps are passing from the cell cultures and offline tumor samples to immediate intraoperative monitoring first of tumor samples and analyzing the quantitative PpIX matrix color coded images with the effective tumor cell invasion.

## Data Availability

The datasets generated for this study are available on request to the corresponding author.

## Ethics Statement

The Ethic Committee of the Canton Ticino approved animal experiments in the animal application TI-05-19 Numero e-TV: 27255.

## Author Contributions

MR conceived the idea and wrote and edited the manuscript. DP performed experimental work and manuscript editing and reviewing. MW, CH, and RG performed Cp9 design and manuscript revision. FM and MD performed experimental work and manuscript editing and reviewing.

### Conflict of Interest Statement

MW, CH, and RG are employed by Carl Zeiss Meditec AG, Oberkochen Germany and declare no further competing interests. The remaining authors declare that the research was conducted in the absence of any commercial or financial relationships that could be construed as a potential conflict of interest.

## Supplementary Material

Video S1 5-ALA guided surgery with OPMI PENTERO 900. Video showing the surgical intervention of glioblastoma removal. Under the blue light the tumor bulk is red, the fluorescence in the tumor margin is pink and more vague.
