# Corrigendum: Quantitative Modulation of PpIX Fluorescence and Improved Glioma Visualization

**DOI:** 10.3389/fsurg.2020.00014

**Published:** 2020-04-02

**Authors:** Michael Reinert, Deborah Piffaretti, Marco Wilzbach, Christian Hauger, Roland Guckler, Francesco Marchi, Maria Luisa D'Angelo

**Affiliations:** ^1^Laboratory for Biomedical Neurosciences, Neurocenter of Southern Switzerland, Ente Ospedaliero Cantonale, Torricella-Taverne, Switzerland; ^2^Department of Neurosurgery, Neurocenter of Southern Switzerland, Ente Ospedaliero Cantonale, Lugano, Switzerland; ^3^Faculty of Biomedical Neurosciences, Università Della Svizzera Italiana, Lugano, Switzerland; ^4^Medical Faculty, University of Bern, Bern, Switzerland; ^5^Faculty of Medicine, Graduate School for Cellular and Biomedical Sciences, University of Bern, Bern, Switzerland; ^6^Carl Zeiss Meditec AG, Oberkochen, Germany

**Keywords:** GBM—glioblastoma multiforme, 5-ALA=5-aminolevulinic acid, protoporphyin IX, quantification, breakdown, visualization, microscope

In the published article there is an error in Figure 4. In the images the third and fifth column of the first row are the same. The image in the third column of the first row (Genistein 25 μM) has been corrected. The image in the fifth column of the first row (DFO 100 μM + Genistein 25 μM) remains as it is.

**Figure 4:**
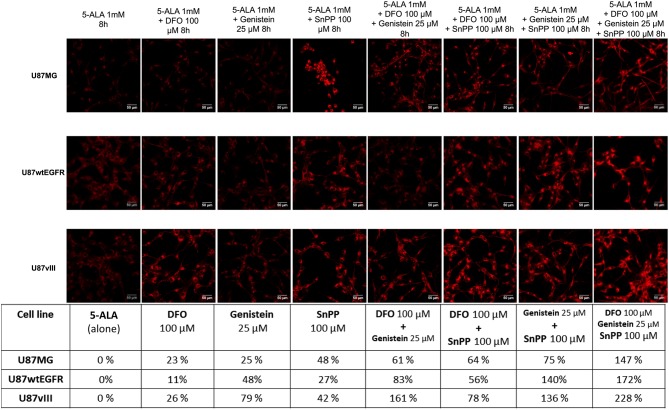
PpIX fluorescence accumulation after single and combined treatments. Confocal images showing the increment in PpIX fluorescence (represented in red, excitation 405 nm and emission 635 nm) in GBM cells after single and combined treatment with two or three drugs compared to 5-ALA alone (represented as 0%). Scale bars represent 50 μm. Table summarizes the increment of PpIX fluorescence in percentage. DFO (deferoxamine), SnPP (tin protoporphyrin IX).

The authors apologize for these errors and state that this does not change the scientific conclusions of the article in any way. The original article has been updated.

